# Virtual reality‐based assessment of cognitive‐locomotor interference in healthy young adults

**DOI:** 10.1186/s12984-021-00834-2

**Published:** 2021-03-22

**Authors:** Anne Deblock-Bellamy, Anouk Lamontagne, Bradford J. McFadyen, Marie-Christine Ouellet, Andreanne K. Blanchette

**Affiliations:** 1 Center for interdisciplinary research in rehabilitation and social integration (Cirris), CIUSSS de la Capitale-Nationale, 525 Boulevard Wilfrid-Hamel, Quebec City, QC G1M 2S8 Canada; 2 Faculty of Medicine, Universite Laval, 1050 Avenue de la Medecine, Quebec City, QC G1V 0A6 Canada; 3 Centre for Interdisciplinary Research in Rehabilitation of Greater Montreal (CRIR), Jewish Rehabilitation Hospital, CISSS de Laval, 3205 Alton-Goldbloom Place, Laval, QC H7V 1R2 Canada; 4 School of Physical and Occupational Therapy, McGill University, 3654 Prom Sir-William-Osler, Montreal, QC H3G 1Y5 Canada; 5 Department of Rehabilitation, Universite Laval, 1050 Avenue de la Medecine, Quebec City, QC G1V 0A6 Canada; 6 Faculty of Social Sciences, School of Psychology, Universite Laval, 2325 rue des Bibliothèques, Quebec City, QC G1V 0A6 Canada

**Keywords:** Dual‐task cost, Locomotion, Cognition, Virtual reality, Healthy adults

## Abstract

**Background:**

A recent literature review emphasized the importance of assessing dual-task (DT) abilities with tasks that are representative of community ambulation. Assessing DT ability in real-life activities using standardized protocols remains difficult. Virtual reality (VR) may represent an interesting alternative enabling the exposure to different scenarios simulating community walking. To better understand dual-task abilities in everyday life activities, the aims of this study were (1) to assess locomotor and cognitive dual-task cost (DTC) during representative daily living activities, using VR, in healthy adults; and 2) to explore the influence of the nature and complexity of locomotor and cognitive tasks on DTC.

**Methods:**

Fifteen healthy young adults (24.9 ± 2.7 years old, 8 women) were recruited to walk in a virtual 100 m shopping mall corridor, while remembering a 5-item list (DT condition), using an omnidirectional platform and a VR headset. Two levels of difficulty were proposed for the locomotor task (with vs. without virtual agent avoidance) and for the cognitive task (with vs. without items modification). These tasks were also performed in single task (ST) condition. Locomotor and cognitive DTC were measured by comparing performances in ST and DT conditions. Locomotor performance was characterized using walking speed, walking fluidity, and minimal distance between the participant and the virtual agent during avoidance. Cognitive performance was assessed with the number of items correctly recalled. Presence of DTC were determined with one-sample Wilcoxon signed-rank tests. To explore the influence of the tasks’ complexity and nature on DTC, a nonparametric two-way repeated measure ANOVA was performed.

**Results:**

No locomotor interference was measured for any of the outcomes. A cognitive DTC of 6.67% was measured (p = .017) while participants performed simultaneously both complex locomotor and cognitive tasks. A significant interaction between locomotor task complexity and cognitive task nature (p = .002) was identified on cognitive DTC.

**Conclusions:**

In challenging locomotor and cognitive conditions, healthy young adults present DTC in cognitive accuracy, which was influenced by the locomotor task complexity task and the cognitive task nature. A similar VR-based protocol might be used to investigate DT abilities in older adults and individuals with a stroke.

## Background

In everyday life activities, people are frequently engaged in situations involving the concurrent execution of locomotor and cognitive tasks, i.e. dual task (DT). Several studies have demonstrated that performing a cognitive task while walking may result in performance deterioration in one or both tasks [1–4]. Different theories suggest that dual-task interferences (DTI) may occur as a result of attentional limitations, but it may reflect a broad variety of underlying mechanisms or processes. The *central bottleneck theory* postulates that central processes might operate sequentially; processing for the second task must consequently be postponed [5]. From a different perspective, the *central capacity sharing theory* suggests that both tasks must share, in parallel, the limited processing capacity available [6, 7].

In most studies documenting DT performances, interference in at least one of the executed tasks was observed, regardless of the studied population [8–11]. However, personal factors seem to influence DTI magnitude. For instance, age-related sensorimotor and cognitive decline, as well as neurological lesions are known to have a detrimental impact on performance when multiple tasks are executed simultaneously [12–16]. Older adults tend to present greater locomotor and/or cognitive performance decrements in dual-task conditions than younger adults, but smaller decrements than age-matched persons with neurological disorders [12].

In addition to personal factors, the complexity of the locomotor task and the nature of the cognitive task might have an impact on the magnitude of the performance decrements [1, 8, 12, 13, 17–22]. For instance, some studies observed that DT cognitive performances were worse during walking tasks involving obstacle avoidance than during simple locomotor tasks [1, 18]. Furthermore, in a meta-analysis, Al-Yahya et al. [12] suggested that cognitive tasks involving internal interfering factors (e.g. mental tracking) seem to disturb walking speed and cadence more than those involving external interfering factors (e.g. reaction time). These observations highlighted the necessity of taking into account the nature and complexity of both locomotor and cognitive tasks for an accurate understanding and interpretation of the DTI.

Dual-task phenomenon was extensively documented, but only a few studies have used cognitive and locomotor tasks representing daily activities. Indeed, commonly used cognitive tasks are adapted from traditional neuropsychological assessments, such as the Stroop test or serial subtractions [23]. Existing literature reflects upon the ecological validity of those executive function assessments. Those assessments seemed inconsistent with the executive functions solicited when performing daily activities [24]. Regarding the locomotor task, participants were most frequently asked to walk forward over a short distance, without any mobile obstacles in most dual-task studies [12, 23].

Moreover, it is important to consider the potential impact of the environment in dual-task assessment. Recent studies have observed differences in locomotor performance between a real-world environment, with high level of distractors, and a quiet hallway, with low level of distractors [25, 26]. Indeed, walking in the community represents a complex activity requiring physical abilities, such as minimal speed, endurance, as well as the ability to negotiate physical environmental demands [27–29]. Everyday community mobility is also known to solicit cognitive functions, especially executive functions and attention [3]. Given the influence of the executed tasks and the environment on DT performance, DT assessment while walking should be performed using cognitive and locomotor tasks that are representative of community ambulation in everyday life.

However, it is difficult to assess dual-task ability, in real-life activities and environment, using standardized and replicable protocols. To overcome this issue, virtual reality (VR) represents an interesting alternative in order to expose individuals with disabilities to different scenarios simulating community walking [30]. The acceptability and feasibility of using VR-based assessment and training have been previously demonstrated in diverse populations with physical limitations [30–32]. In regard to dual-task assessment, this technology may enable the development of standard DT assessment protocols in meaningful simulated environments [30]. Moreover, difficulty of the tasks and environmental distractors can be controlled in VR [33]. Given the latest advancements in technology, some VR systems are now low-cost and easy to use facilitating their adoption in clinical practice.

The aims of this study were: (1) to assess locomotor and cognitive dual-task costs in activities that are representative of daily living, using virtual reality, in healthy adults; and (2) to explore the influence of the nature and complexity of cognitive and locomotor tasks on DTC.

## Methods

### Participants

Healthy adults were recruited, from the university student population, using a convenience sample. Participants were excluded if they reported unstable health conditions or any deficits that could affect performance during the experiment. All participants provided written informed consent prior to their participation in the study. This study was approved by the institutional ethics review board of the *Centre intégré universitaire de santé et de services sociaux de la Capitale-Nationale* (# 2019 − 1720).

### General procedure

In this cross-sectional study, participants were assessed over one session at the Centre for Interdisciplinary Research in Rehabilitation and Social Integration (Cirris). Before the experiment, each participant completed a sociodemographic questionnaire (age, sex, occupation). The experiment session began with a period of familiarization with the equipment and the VR environment. In each trial, participants had to walk through a pathway (approximately 130m) including direction changes and virtual agents to avoid. The familiarization period continued until the stabilization of trial duration (less than 10% of difference between trials).

### Experimental setup

**Fig. 1:**
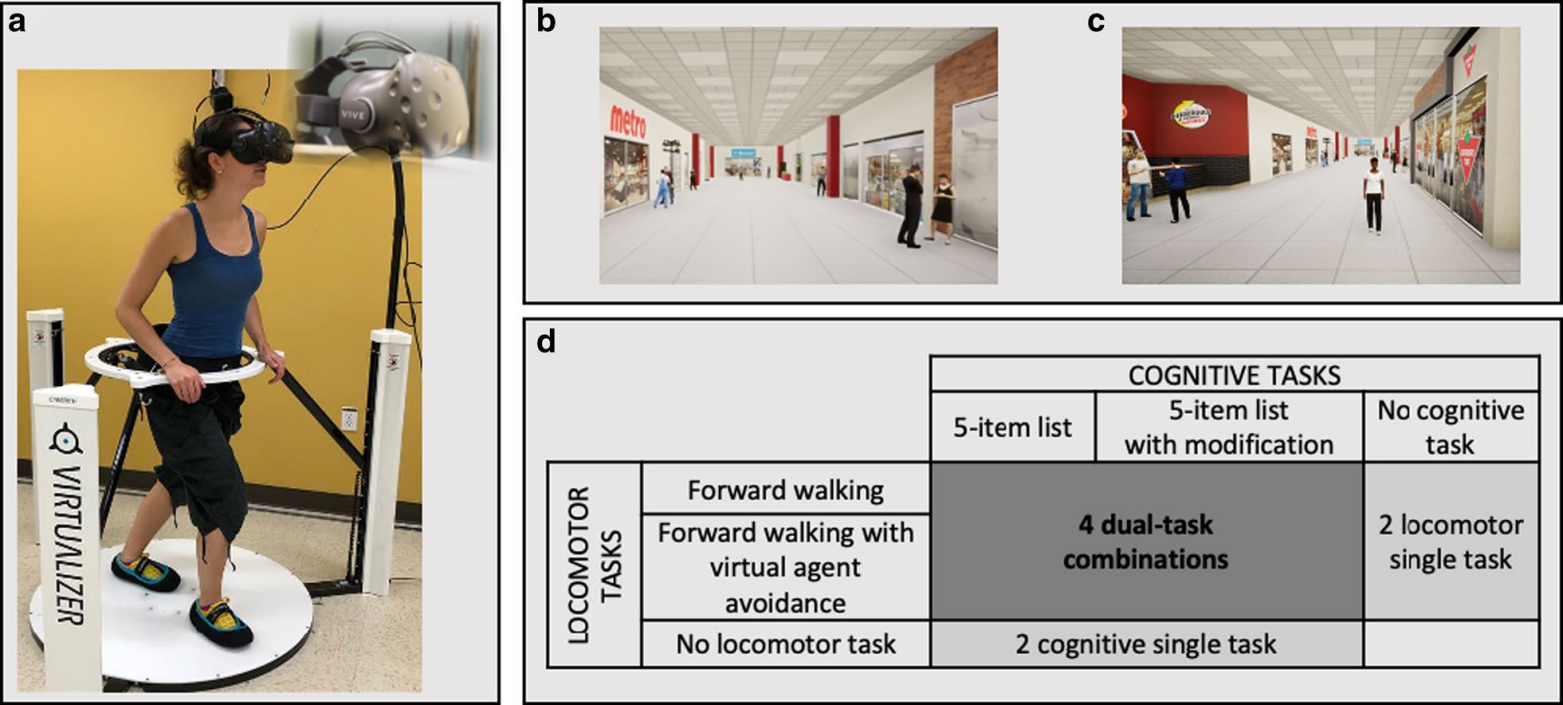
Experimental setup. **a** Omnidirectional platform (Virtualizer, Cyberith GmbH) and virtual reality headset (Vive, HTC); **b** virtual environment without virtual agent avoidance; **c** virtual environment with virtual agent avoidance; **d** description of the 8 experimental tasks

A VR setup was used to assess DT ability in a virtual shopping mall corridor, where participants had to walk while remembering a shopping list. Equipment included an omnidirectional platform (Virtualizer, Cyberith GmbH, Vienna, Austria) and a VR headset (Vive, HTC Corporation) with a field of view of 110° and a refresh rate of 90Hz. The omnidirectional platform provided the means to walk in the virtual scene, while remaining on the spot in the physical environment [34]. It consisted of a low-friction baseplate and a rotatable ring at the pelvic level equipped with a harness. The system included seven optical motion sensors. Six were imbedded in the baseplate to track foot movements and one was imbedded in the ring to track the orientation of the participant. Signals from these sensors enabled the participants to progress, in all directions (360 degrees in the horizontal plane), in the virtual environment. In combination with the signals from the ring sensor, headset motion sensors allowed visualization of the virtual environment with respect to head orientation. For security reasons, participants were installed in a harness fixed to the platform ring, but no weight support was provided. Even if the ring was vertically mobile, there was a lower mechanical stop set for each participant in order to avoid falls on the platform. Participants also had the possibility to lightly grab the anterior part of the ring with their hands, if needed (Fig. 1a).

### Virtual environment

All experimental tasks were performed in a virtual 100m straight shopping mall corridor. The environment included virtual agents (mobile or stationary) that did not interfere with participants’ walking trajectory (visual distractors only), and which were placed on either side of the participants’ walking path (Fig. 1b). Participants were exposed to several scenes of a 100m shopping mall corridor, with different configurations (Fig. 1b, c). Shopping mall ambient noises were played through a speaker located within 1m of the participant.

### Experimental tasks

Locomotor and cognitive tasks with two levels of difficulty were used (Fig. 1d). Participants performed locomotor and cognitive tasks separately (single task; ST) and simultaneously (DT) in the virtual environment.

#### Single locomotor tasks

**Fig. 2:**
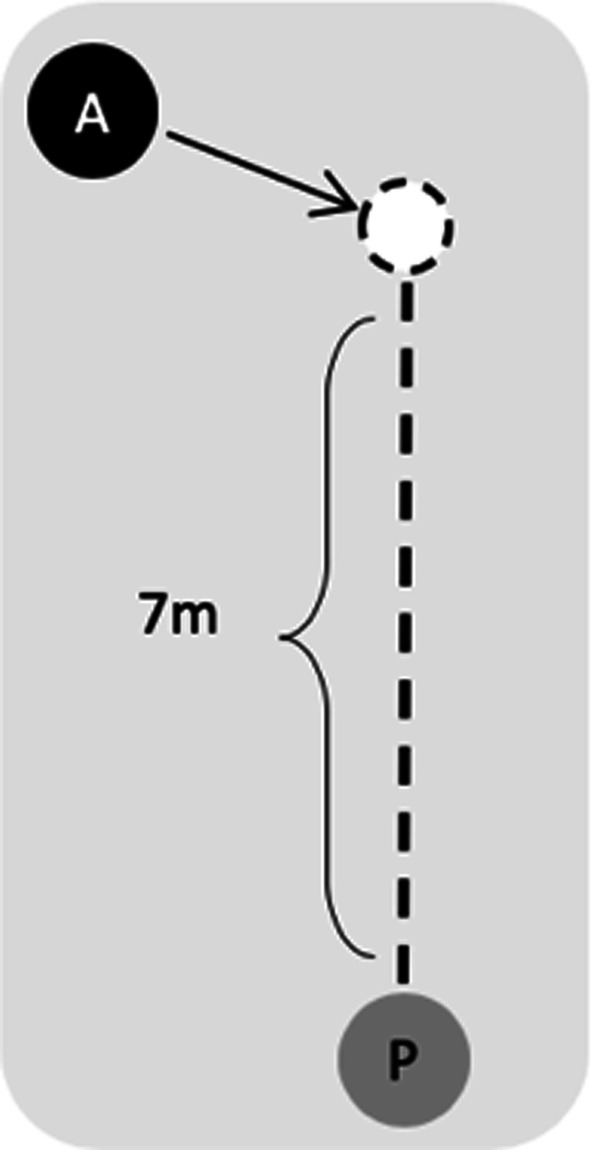
Virtual agent trajectory. Once the non-visible target (white circle), located at 7 meters in front of the participant, was hit, the virtual agent (A) walked toward the participant (P)

- Forward walking: Participants had to walk straight ahead to a store located at the end of the corridor, at their comfortable speed.
- Forward walking with virtual agent avoidance (complex locomotor task): As participants walked to the store, they had to avoid three female virtual agents approaching from different directions (Fig. 1c). The virtual agents, placed at 30 m apart from each other, were programmed to reach, one at time, a non-visible point fixed at 7 m in front of the participants’ real-time position (within the sagittal plane). The virtual agents walked at participants’ speed or at 1.2 m/s if participants were slower. Once the virtual agents arrived at the fixed point in front of the participant, they were programmed to walk toward the participant along that line, without considering further lateral displacements of the participant (Fig. 2).

For all the locomotor tasks, the end of the pathway was indicated by an orange circle located on the floor, which turned green as soon as it was reached by the participants.

### Single cognitive tasks

In ST condition, participants performed the cognitive task while sitting in front of a store in the virtual shopping mall.

- 5-item shopping list: Participants had to listen to and remember a 5-item shopping list, repeated twice. After a predetermined period of time (based on the maximal duration of DT conditions), participants had to verbally identify each of the items.
- 5-item shopping list with modification (complex cognitive task): After listening to and remembering an initial 5-item shopping list, participants had to take into account the modification of two items delivered in an audio message repeated twice. This new message can be heard at a predetermined moment, corresponding to approximately one third of the time taken in DT conditions. It should be noted that the non-modified items were not repeated. At the end of the task, participant had to verbally identify the 5 items while taking into account the modification.

The 4 ST and the 4 DT combinations were performed following a pseudo-random sequence and this sequence was then repeated three times, for a total of 24 trials. In order to standardize the retention time across conditions, cognitive single tasks were performed at the end of each sequence. Thus, the duration of these trials was based on the maximal duration of DT conditions using the same cognitive task difficulty. In DT conditions, the initial list of items was delivered after 2 m of walking and participants had to verbally identify the remembered items at the end of the locomotor task. Before each trial, no information about the condition (ST or DT) or the difficulty of the task(s) were shared with the participant. For the DT conditions, no prioritization instruction was given.

### Data collection

Locomotor performances were quantified with the *walking speed and fluidity* as well as with the *minimal distance between the participant and virtual agents*, for the conditions involving virtual agent avoidance. Participants’ displacements along antero-posterior and medio-lateral axes were analyzed, based on the signals of the platform sensors (baseplate and ring). The position data of the participants and virtual agents were filtered with a fourth-order Butterworth filter (low pass with 6Hz cut-off frequency). *Walking speed* was derived from the participant position. *Walking fluidity* was quantified by the number of acceleration zero-crossings. Walking acceleration was obtained from the second derivation of the positions and filtered with a fourth-order Butterworth filter with a 2Hz cutoff frequency. Cognitive performance was measured with the number of items correctly recalled, with a maximal score of 5 for each condition.

Data processing was performed using custom-made scripts written in MATLAB R2018b (The MathWorks, Inc., Natick, Massachusetts, USA) and Microsoft Excel 16.24 (Microsoft Corporation, Redmond, Washington, USA).

### Data analyses

For each dependent variable, dual-task costs (DTC) were calculated using the mean of 3 trials, with the following formula:

$$ \frac{(Single{\text{-}}task\; perfomance) - (dual{\text{-}}task\; perfomance)}{Mean\; Single{\text{-}}task\; perfomance}*100$$

A positive DTC in walking speed indicates a lower speed in the DT condition, while a negative DTC for walking fluidity indicated a worse fluidity (less zero-crossings) in the DT condition. A positive DTC in minimal distance and in cognitive accuracy meant, respectively, a smaller minimal distance between the participant and the virtual agents and a worse cognitive performance in DT conditions than in ST conditions.

### Statistical analyses

Parametric descriptive statistics (mean and standard deviation (SD)) were used to characterize participants. Given the fact that the data did not follow a normal distribution, nonparametric descriptive statistics (median and 25th–75th percentiles of all participants) were used to describe DTC observed in each DT combination. For the first objective, one sample Wilcoxon signed-rank tests were then used to determine whether the medians were equal to zero. To explore the influence of the tasks’ complexity and nature on DTC, a nonparametric two-way repeated measure ANOVA in factorial experiments (ANOVA-type statistic—ATS, Nonparametric Analysis of Longitudinal Data in Factorial Experiments—NparLD [35]) was performed on DTC that were significantly different from zero. If significant interactions were observed, post-hoc analyses were performed with one-way nparLD. Significance level was set at 0.05.

Descriptive statistics were performed using IBM SPSS Statistics 25.0 (SPSS Inc., Chicago, Illinois, USA) and NparLD package was used to perform the nonparametric two-way repeated measure ANOVA using the R software (R Studio 1.2, Inc., USA).

## Results

Fifteen healthy young adults were recruited (24.9 ± 2.7 years old; 8 women). All participants completed the entire experimental protocol. Due to technical issues, data was missing for one trial in one participant (2 trials were averaged in instead of 3 for the performance during DT condition combining both simple locomotor and cognitive tasks).

Locomotor and cognitive DTC for each outcome and each DT combination are described in Table 1. From a global perspective, changes in performance when participants executed locomotor and cognitive tasks simultaneously were small, as demonstrated by DTC ranging from − 2.60 to 6.67%. While no significant locomotor DTC was observed (*p*-values ranging between 0.842 and 0.078), a significant cognitive DTC was measured when complex locomotor and complex cognitive tasks were combined. Indeed, a positive cognitive DTC (6.67%; *p* = .017) was highlighted, which means that complex cognitive task performance deteriorated when participants avoided virtual agents compared to their performance in simple locomotor task.

**Table 1:** Median DTC (25th-75th percentiles) for each outcome and each dual-task combination

	Cognitive tasks				
		5-item list		5-item list with modification	
		DTC	*p*-value	DTC	*p*-value
Locomotor tasks	**Forward walking**				
	Walking speed	− 2.60% (− 4.56; 1.42)	.394	− 1.04% (− 4.49; 2.98)	.570
	Walking fluidity	1.72% (− 2.55; 4.75)	.345	1.27% (− 3.72; 2.96)	.842
	Cognitive accuracy	0.0% (0.0; 0.0)	.157	0.0% (0.0; 0.0)	.916
	**Forward walking with virtual agents**				
	Walking speed	2.79% (− 1.27; 5.28)	.078	3.39% (− 2.47; 4.88)	.211
	Walking fluidity	− 2.03% (− 4.29; 1.20)	.100	0.0% (− 7.98; 3.56)	.433
	Minimal distance	0.54% (− 5.81; 5.64)	.650	− 1.31% (− 9.18; 2.75)	.211
	Cognitive accuracy	0.0% (0.0; 0.0)	.083	6.67% (0.0; 7.69)	.*017*

Significant *p* values are in italic

Since the only significant DTC was found in the cognitive accuracy, the influence of tasks’ nature and complexity was explored exclusively on this outcome (Table 2). A significant interaction between locomotor and cognitive task complexity (ATS = 9.48; *p* = .002), as well as a significant main effect of locomotor task complexity (ATS = 7.15; *p* = .007) were identified. Post-hoc tests indicated that, during complex locomotor tasks, cognitive accuracy DTC was larger when (*p* = .001). Furthermore, cognitive accuracy DTC was larger during a complex locomotor task than during a simple locomotor task while performing a complex cognitive task (*p* = .004). This result indicated that the cognitive accuracy DTC were larger if both complex cognitive and locomotor tasks were performed simultaneously in comparison of conditions involving a simple task. Similarly, no significant difference in cognitive accuracy DTC was identified between complex and simple locomotor tasks when participants performed a simple cognitive task (*p* = .570), nor between complex and simple cognitive tasks when participants performed a simple locomotor task (*p* = .590).

**Table 2:** Influence of tasks’ nature and complexity on DTC

	Main effect		Interaction	Post-hoc tests
	Locomotor task complexity	Cognitive task complexity	Locomotor task complexity*cognitive task complexity	
Cognitive accuracy DTC	ATS = 7.15 *p* = .*007*	ATS = 2.20 *p* = .138	ATS = 9.48 *p* = .*002*	**Complex locomotor task conditions** Cognitive DTC during complex cognitive task ≠ cognitive DTC during simple cognitive task (*p* = .*001*) **Complex cognitive task conditions** Cognitive DTC during complex locomotor task ≠ cognitive DTC during simple locomotor task (*p* = .*004*) **Simple locomotor task conditions** Cognitive DTC during complex cognitive task = cognitive DTC during simple cognitive task (*p* = .590) **Simple cognitive task conditions** Cognitive DTC during complex locomotor task = cognitive DTC during simple locomotor task (*p* = .570)

Significant *p* values are in italic

## Discussion

In this present study, DT abilities of healthy young adults were assessed in a virtual environment with locomotor and cognitive tasks of varying complexities and which are representative of daily living activities. DTC was observed only during the condition combining complex locomotor and cognitive tasks. In this specific condition, a detrimental DT effect was observed on cognitive performance, but not on locomotor performance. DTC findings observed across task combinations indicate that the nature and complexity of the tasks influence the magnitude of cognitive accuracy DTC.

Neuropsychological theories of dual-tasking [5–7] may help to understand these results. Considering that no interference was found when participants performed DT involving at least one simple task, it may be hypothesized that central processes were not overloaded during these less complex DT combinations. Indeed, when walking forward, on an even surface without any obstacle, the control of gait is fairly automatic and minimal cognitive processes are involved (reviewed in [36]). Results of the present study showing no interference in all DT combinations involving simple forward walking at comfortable walking speed may therefore be explained by a higher level of gait automaticity. Yet, previous studies have demonstrated that the reliance on executive functions increases as the locomotor task becomes more challenging (e.g., [3, 37]). This contribution of executive functions to obstacle avoidance during walking was also demonstrated through an increased prefrontal cortex activation [38–40]. Thus, executive function decline may contribute to walking disorders [3]. Regarding the cognitive tasks used in the present study, short-term memory was undoubtedly involved when participants listened to and remembered a 5-item shopping list. More complex executive functions, such as cognitive flexibility, attention, and inhibition [41], were solicited when participants had to take into account modifications of this shopping list as they walked. The DTC observed might therefore reflect overloaded attentional circuits when concurrently avoiding obstacles while walking and remember the 5-item list which was modified during the course of the trial. The absence of interference in performances when executing the simple cognitive task (remembering a 5-item shopping list) and the complex locomotor task (walking with obstacle avoidance) suggests that these tasks do not share the same mental processes.

While our results are consistent with the basic assumption of dominant neuropsychological theories suggesting that DT interference results from limited processing capacity, our experimental protocol was not designed to draw conclusions about the mechanisms or processes involved or to support one theory over another.

The absence of significant locomotor DTC might be related to the high variability across participants when looking at their locomotor performances in DT conditions. Participants seemed to use different locomotor strategies to deal with the proposed DT conditions. While some participants decreased their walking speed while performing an additional cognitive task, others increased their speed, as demonstrated by positive and negative DTC, respectively. Increasing walking speed when dual tasking may be surprising and counterintuitive at first, but it may be explained by the participant’s intention to reduce the retention time of the memory task (negative DTC). This strategy has been previously observed when participants were exposed to a DT condition involving a memory task [42]. In contrast, decreasing walking speed (positive DTC) might be considered as a compensatory mechanism used to maintain stability while dual tasking. This strategy was observed in older adults when they were exposed to a more challenging DT condition [43].

Many studies have highlighted the presence of DTC in healthy young adults [1, 8, 15, 18, 19, 44]. Contrarily to the results of the present experiment, interference in locomotor performance was obtained in several studies, regardless of the executed locomotor tasks (forward walking [1, 8, 15, 44], walking with obstacles [19, 45, 46]). Interestingly, all studies except one [19] involved a cognitive task in which participants had to provide a verbal response (e.g. spontaneous speech, serial subtractions, auditory Stroop test). Most studies interpret the performance on fluency tests as an indicator of cognitive function and do not consider their motor demands [12]. However, verbal cognitive tasks should be considered as cognitive-motor tasks, since they necessitate phonarticulatory coordination, in addition to response inhibition and phonological processing [47]. Yardley et al. [48] have underlined the importance of considering both aspects of verbal tasks. Indeed, they observed that postural instability while performing an arithmetic task was principally induced by the perturbing effects of articulating sounds, rather than the attentional demands. Instability might be the result of a central interference between central motor structures involved in the speech production and in balance control [48]. Thus, the locomotor DT interferences observed in studies using verbal cognitive tasks during walking might be explained by increased motor demands. In contrast, the protocol used in the present study necessitated that participants provide a verbal response (recall of items) once the locomotor task was completed, which may have led to divergent results with the previously mentioned studies.

The presence of a cognitive DTC without locomotor DTC during the complex locomotor task may also be explained by the phenomenon of prioritization in DT conditions. Yogev-Seligmann et al. [49] proposed an integrated model that provides theoretical foundation to explain prioritization strategies during walking while dual tasking. According to this model, without specific instructions on prioritization, healthy young adults focus on the cognitive task as long as the risk of falling while walking remains low but shift their attention toward the locomotion task when it becomes more challenging, in order to ensure their safety. In the present study, no DTC was induced when participants had to perform a cognitive task while simply walking forward (without virtual agents to avoid). Task prioritization was therefore not necessary. Deterioration in cognitive performance but not in locomotor performance may however indicate that participants have prioritized locomotion when they were asked to perform a complex cognitive task while walking and avoiding collisions with virtual agents. This finding is consistent with the integrated model of task prioritization [49]. Considering that participants were immersed in a virtual environment that simulated a shopping mall, without any sensory feedback from the real environment, they may have perceived virtual agent avoidance while walking as a real threatning condition. Previous study have provided evidence of the implementation of circumvention strategies in a virtual environment that are comparable to those in the physical environment [50]. The small differences observed in obstacle circumvention outcomes between environments were explained by the use of safer or more conservative strategies, therefore suggesting that participants did not feel completely stable and safe when avoiding obstacles in the virtual environment.

## Study limitations

Walking on an omnidirectional platform has an impact on spatiotemporal parameters, lower limb kinematics and muscle activation pattern compared to overground walking [51]. Participants had to push their hips against the ring and slide above the surface; while one foot slides backward, the other foot steps forward [34]. Higher muscle activation amplitudes in several lower limb muscle groups were observed when young adults walked on an omnidirectional platform vs. overground [51]. This newly learned walking pattern may require greater attentional demands for task execution and performance than overground walking. In order to optimize learning of the locomotor task, participants had to complete a familiarization period prior to the experiment. While familiarization was completed within a reasonably short period of time in healthy young adults, we expect this crucial step to be longer in older adults and individuals with neurological disorders. For this reason, the experimental protocol will be adapted to include two session of familiarization with a delay between sessions, which is likely to help in motor skill consolidation.

The enhanced somatosensory inputs provided by the ring and the harness may also facilitate perception of movement and consequently increase dynamic stability during walking. In previous studies, the effect of enhanced sensory feedback while walking has been investigated through the use of haptic tools or specialized footwear (e.g. vibrating or textured insoles) [52–54]. These techniques have induced modifications in walking behavior, including an increase in whole body stability, as well as a reduction in variability of gait step parameters and lower limb muscle activity [55]. Even if such effects of haptic feedback might be present while walking on the omnidirectional platform, they were not large enough to attenuate the larger levels of muscle activation amplitude observed when walking on the omnidirectional platform compared to overground walking [51]. Considering that sensory feedback may help to drive the active motoneurons [56], enhanced reliable somatosensory information may facilitate the automaticity of walking. A neurophysiological study using functional near-infrared spectroscopy has demonstrated that enhanced somatosensory feedback during walking reduced metabolic activity of the prefrontal cortex, which is consistent with lower utilization of attentional processing resources during walking [57].

Since the same equipment was used in both single- and dual-task conditions, it is unlikely that these differences affect DTC results and conclusions. However, it may have an influence on the interpretation of results, especially when a parallel is drawn with DT abilities during overground walking.

## Conclusions and future directions

While performing a cognitive task while walking in a virtual environment, locomotor-related cognitive interference was observed in healthy young adults when the executed tasks (cognitive and locomotor) were both complex. No DTC was observed in any other conditions, indicating that the complexity and the nature of the tasks influenced the magnitude of the cognitive interference. Considering that community walking is frequently identify as a meaningful rehabilitation goal for persons with walking limitations, assessing DT ability in activities that are representative of daily living is crucial. The VR assessment protocol described in the present study will be used in future studies to characterize DT abilities of healthy older adults and individuals who sustained a stroke.

## Data Availability

The datasets generated and analysed during the current study are available from the corresponding author on reasonable request.

## Abbreviations

ATS: ANOVA-type statistic
DT: Dual task
DTC: Dual-task cost
DTI: Dual-task interference
Hz: Hertz
m: Meter
m/s: Meters per second
NparLD: Nonparametric analysis of longitudinal data in factorial experiments
*p*: *p*-value
SD: Standard deviation
ST: Single task
VR: Virtual realty

## References

[1] Raffegeau TE, Haddad JM, Huber JE, Rietdyk S (2018). Walking while talking: Young adults flexibly allocate resources between speech and gait. Gait Posture.

[2] Sigman M, Dehaene S (2008). Brain mechanisms of serial and parallel processing during dual-task performance. J Neurosci.

[3] Yogev-Seligmann G, Hausdorff JM, Giladi N (2008). The role of executive function and attention in gait. Movement Disord.

[4] Woollacott M, Shumway-Cook A (2002). Attention and the control of posture and gait: a review of an emerging area of research. Gait Posture.

[5] Pashler H (1994). Dual-task interference in simple tasks: data and theory. Psychol Bull.

[6] Tombu M, Jolicœur P (2003). A central capacity sharing model of dual-task performance. J Exp Psychol Hum Percept Perform.

[7] Tombu M, Jolicœur P (2005). Testing the predictions of the central capacity sharing model. J Exp Psychol Hum Percept Perform.

[8] Patel P, Bhatt T (2014). Task matters. Influence of different cognitive tasks on cognitive-motor interference during dual-task walking in chronic stroke survivors. Topics Stroke Rehabil.

[9] Plummer P, Eskes G, Wallace S, Giuffrida C, Fraas M, Campbell G (2013). Cognitive-motor interference during functional mobility after stroke: state of the science and implications for future research. Archiv Phys Med Rehabil.

[10] Smith E, Cusack T, Cunningham C, Blake C (2017). The influence of a cognitive dual task on the gait parameters of healthy older adults: a systematic review and meta-analysis. J Aging Phys Activity.

[11] Tramontano M, Morone G, Curcio A, Temperoni G, Medici A, Morelli D (2017). Maintaining gait stability during dual walking task: effects of age and neurological disorders. Eur J Phys Rehabil Med.

[12] Al-Yahya E, Dawes H, Smith L, Dennis A, Howells K, Cockburn J (2011). Cognitive motor interference while walking: a systematic review and meta-analysis. Neurosci Biobehav Rev.

[13] Gaillardin F, Baudry S (2018). Influence of working memory and executive function on stair ascent and descent in young and older adults. Exp Gerontol.

[14] Seidler RD, Bernard JA, Burutolu TB, Fling BW, Gordon MT, Gwin JT (2010). Motor control and aging: links to age-related brain structural, functional, and biochemical effects. Neurosci Biobehav Rev.

[15] Yogev-Seligmann G, Rotem-Galili Y, Mirelman A, Dickstein R, Giladi N, Hausdorff JM (2010). How does explicit prioritization alter walking during dual-task performance? Effects of age and sex on gait speed and variability. Phys Ther.

[16] McFadyen BJ, Gagné M-È, Cossette I, Ouellet M-C (2017). Using dual task walking as an aid to assess executive dysfunction ecologically in neurological populations: a narrative review. Neuropsychological Rehabilitation..

[17] Maclean LM, Brown LJE, Khadra H, Astell AJ (2017). Observing prioritization effects on cognition and gait: The effect of increased cognitive load on cognitively healthy older adults’ dual-task performance. Gait Posture.

[18] Siu K-C, Chou L-S, Mayr U, van Donkelaar P, Woollacott MH (2008). Does inability to allocate attention contribute to balance constraints during gait in older adults?. J Gerontol A Biol Sci Med Sci.

[19] Souza Silva W, McFadyen B, Fung J, Lamontagne A (2019). Effects of age on obstacle avoidance while walking and deciphering text versus audio phone messages. Gerontology.

[20] Wrightson JG, Ross EZ, Smeeton NJ (2016). The effect of cognitive-task type and walking speed on dual-task gait in healthy adults. Motor Control.

[21] Aravind G, Lamontagne A (2017). Dual tasking negatively impacts obstacle avoidance abilities in post-stroke individuals with visuospatial neglect: task complexity matters!. Restorat Neurol Neurosci.

[22] Cossette I, Ouellet M-C, McFadyen BJ (2014). A preliminary study to identify locomotor-cognitive dual tasks that reveal persistent executive dysfunction after mild traumatic brain injury. Archiv Phys Med Rehabil.

[23] Deblock-Bellamy A, Lamontagne A, Blanchette AK (2020). Cognitive-locomotor dual-task interference in stroke survivors and the influence of the tasks: a systematic review. Front Neurol.

[24] Chaytor N, Schmitteredgecombe M, Burr R (2006). Improving the ecological validity of executive functioning assessment. Archiv Clin Neuropsychol.

[25] Plummer P, Altmann L, Feld J, Zukowski L, Najafi B, Giuliani C (2020). Attentional prioritization in dual-task walking: effects of stroke, environment, and instructed focus. Gait Posture.

[26] Zukowski LA, Feld JA, Giuliani CA, Plummer P (2019). Relationships between gait variability and ambulatory activity post stroke. Topics Stroke Rehabil.

[27] Balasubramanian CK, Clark DJ, Fox EJ (2014). Walking adaptability after a stroke and its assessment in clinical settings. Stroke Res Treat.

[28] Patla AE, Shumway-Cook A (1999). Dimensions of mobility: defining the complexity and difficulty associated with community mobility. J Aging Phys Activity.

[29] Shumway-Cook A, Patla AE, Stewart A, Ferrucci L, Ciol MA, Guralnik JM (2002). Environmental demands associated with community mobility in older adults with and without mobility disabilities. Phys Ther.

[30] Camara Lopez M, Deliens G, Cleeremans A (2016). Ecological assessment of divided attention: what about the current tools and the relevancy of virtual reality. Revue Neurol.

[31] Robitaille N, Jackson PL, Hébert LJ, Mercier C, Bouyer LJ, Fecteau S (2017). A Virtual Reality avatar interaction (VRai) platform to assess residual executive dysfunction in active military personnel with previous mild traumatic brain injury: proof of concept. Disabil Rehabil Assis Technol.

[32] Freitag F, Brucki SMD, Barbosa AF, Chen J, Souza C, Valente DF (2019). Is virtual reality beneficial for dual-task gait training in patients with Parkinson’s disease?&nbsp;A systematic review. Dement Neuropsychol.

[33] Rizzo AA, Schultheis M, Kerns KA, Mateer C (2004). Analysis of assets for virtual reality applications in neuropsychology. Neuropsychol Rehabil.

[34] Cakmak T, Hager H. Cyberith virtualizer: a locomotion device for virtual reality. In: ACM SIGGRAPH 2014 Emerging Technologies on - SIGGRAPH ’14 [Internet]. Vancouver, Canada: ACM Press; 2014. Accessed 10 Jul 2020. p. 1–1. http://dl.acm.org/citation.cfm?doid=2614066.261410510.1145/2614066.2614105.

[35] Noguchi K, Gel YR, Brunner E, Konietschke F, Npar LD. An R software package for the nonparametric analysis of longitudinal data in factorial experiments. J Stat Soft. 2012;50:12. 10.18637/jss.v050.i12.

[36] Clark DJ (2015). Automaticity of walking: functional significance, mechanisms, measurement and rehabilitation strategies. Front Hum Neurosci..

[37] Ble A, Volpato S, Zuliani G, Guralnik JM, Bandinelli S, Lauretani F (2005). Executive function correlates with walking speed in older persons: The InCHIANTI Study: executive function is associated with walking speed. J Am Geriatr Soc.

[38] Clark DJ, Rose DK, Ring SA, Porges EC (2014). Utilization of central nervous system resources for preparation and performance of complex walking tasks in older adults. Front Aging Neurosci..

[39] Maidan I, Nieuwhof F, Bernad-Elazari H, Reelick MF, Bloem BR, Giladi N (2016). The role of the frontal lobe in complex walking among patients with Parkinson’s disease and healthy older adults: an fNIRS Study. Neurorehabil Neural Repair..

[40] Maidan I, Shustak S, Sharon T, Bernad-Elazari H, Geffen N, Giladi N (2018). Prefrontal cortex activation during obstacle negotiation: what’s the effect size and timing?. Brain Cognition..

[41] Diamond A (2013). Executive functions. Annu Rev Psychol..

[42] Kizony R, Levin MF, Hughey L, Perez C, Fung J (2010). Cognitive load and dual-task performance during locomotion poststroke: a feasibility study using a functional virtual environment. Phys Ther..

[43] Kelly VE, Schrager MA, Price R, Ferrucci L, Shumway-Cook A (2008). Age-associated effects of a concurrent cognitive task on gait speed and stability during narrow-base walking. J Gerontol Series A..

[44] Kelly VE, Janke AA, Shumway-Cook A (2010). Effects of instructed focus and task difficulty on concurrent walking and cognitive task performance in healthy young adults. Exp Brain Res.

[45] Raffegeau TE, Krehbiel LM, Kang N, Thijs FJ, Altmann LJP, Cauraugh JH, et al A meta-analysis: Parkinson’s disease and dual-task walking. Parkinsonism. & Related Disorder. 2018. https://linkinghub.elsevier.com/retrieve/pii/S135380201830540610.1016/j.parkreldis. Accessed 12 Dec 2018. 10.1016/j.parkreldis.2018.12.012 PMC8487457 30594454

[46] Gérin-Lajoie M, Richards CL, McFadyen BJ (2005). The negotiation of stationary and moving obstructions during walking: anticipatory locomotor adaptations and preservation of personal space. Motor Control..

[47] Barbosa AF, Voos MC, Chen J, Francato DCV, Souza C, de Barbosa O (2017). Cognitive or cognitive-motor executive function tasks? Evaluating verbal fluency measures in people with Parkinson’s Disease. Biomed Res Int.

[48] Yardley L, Gardner M, Leadbetter A, Lavie N (1999). Effect of articulatory and mental tasks on postural control. NeuroReport..

[49] Yogev-Seligmann G, Hausdorff JM, Giladi N (2012). Do we always prioritize balance when walking? Towards an integrated model of task prioritization: integrated Model of Task Prioritization. Mov Disord.

[50] Bühler MA, Lamontagne A (2019). Locomotor circumvention strategies in response to static pedestrians in a virtual and physical environment. Gait Posture.

[51] Soni S, Lamontagne A (2020). Characterization of speed adaptation while walking on an omnidirectional treadmill. J NeuroEng Rehabil.

[52] Hedayat I, Moraes R, Lanovaz JL, Oates AR (2017). Different haptic tools reduce trunk velocity in the frontal plane during walking, but haptic anchors have advantages over lightly touching a railing. Exp Brain Res..

[53] Palluel E, Olivier I, Nougier V (2009). The lasting effects of spike insoles on postural control in the elderly. Behav Neurosci.

[54] Lipsitz LA, Lough M, Niemi J, Travison T, Howlett H, Manor B (2015). A shoe insole delivering subsensory vibratory noise improves balance and gait in healthy elderly people. Arch Phys Med Rehabil.

[55] Oates AR, Hauck L, Moraes R, Sibley KM (2017). The effects of haptic input on biomechanical and neurophysiological parameters of walking: a scoping review. Gait Posture.

[56] Nielsen JB (2003). How we walk: central control of muscle activity during human walking. Neuroscientist.

[57] Clark DJ, Christou EA, Ring SA, Williamson JB, Doty L (2014). Enhanced somatosensory feedback reduces prefrontal cortical activity during walking in older adults. J Gerontol Series A..

